# SNP genotyping to screen for a common deletion in CHARGE Syndrome

**DOI:** 10.1186/1471-2350-6-8

**Published:** 2005-02-14

**Authors:** Seema R Lalani, Arsalan M Safiullah, Susan D Fernbach, Michael Phillips, Carlos A Bacino, Laura M Molinari, Nancy L Glass, Jeffrey A Towbin, William J Craigen, John W Belmont

**Affiliations:** 1 Department of Molecular and Human Genetics, Baylor College of Medicine, Houston, Texas, USA; 2 Genome Quebec and McGill University Innovation Centre, McGill University, Montreal, Quebec, Canada; 3 Department of Anesthesiology, Baylor College of Medicine, Houston, Texas, USA; 4 Department of Pediatrics, Baylor College of Medicine, Houston, Texas, USA; 5 Department of Pediatrics (Cardiology), Baylor College of Medicine, Houston, Texas, USA

## Abstract

**Background:**

CHARGE syndrome is a complex of birth defects including coloboma, choanal atresia, ear malformations and deafness, cardiac defects, and growth delay. We have previously hypothesized that CHARGE syndrome could be caused by unidentified genomic microdeletion, but no such deletion was detected using short tandem repeat (STR) markers spaced an average of 5 cM apart. Recently, microdeletion at 8q12 locus was reported in two patients with CHARGE, although point mutation in *CHD7 *on chromosome 8 was the underlying etiology in most of the affected patients.

**Methods:**

We have extended our previous study by employing a much higher density of SNP markers (3258) with an average spacing of approximately 800 kb. These SNP markers are diallelic and, therefore, have much different properties for detection of deletions than STRs.

**Results:**

A global error rate estimate was produced based on Mendelian inconsistency. One marker, rs431722 exceeded the expected frequency of inconsistencies, but no deletion could be demonstrated after retesting the 4 inconsistent pedigrees with local flanking markers or by FISH with the corresponding BAC clone. Expected deletion detection (EDD) was used to assess the coverage of specific intervals over the genome by deriving the probability of detecting a common loss of heterozygosity event over each genomic interval. This analysis estimated the fraction of unobserved deletions, taking into account the allele frequencies at the SNPs, the known marker spacing and sample size.

**Conclusions:**

The results of our genotyping indicate that more than 35% of the genome is included in regions with very low probability of a deletion of at least 2 Mb.

## Background

CHARGE Association is characterized by ocular coloboma, cranial nerve abnormalities, common outflow tract heart defects, choanal atresia, cupped-shaped pinnae, Mondini dysplasia of the inner ear [1,2] and growth delay. The embryology and mechanisms of maldevelopment in CHARGE are not well understood. CHARGE Association may be genetically heterogeneous, a possibility supported by the rare and variable chromosomal aberrations observed in a few affected individuals. We have identified a *de novo *mutation in a semaphorin gene, *SEMA3E*, in an affected patient, identified upon mapping the translocation breakpoints in an unrelated individual with a *de novo *balanced translocation involving chromosomes 2 and 7: karyotype 46, XY, t(2;7)(p14;q21.11) [3]. Recently, Vissers *et al*. have reported mutations in *CHD7 *gene, a member of the chromodomain family in a substantial number of patients [4]. In a large number of children, however, the genetic mechanism of this complex birth defect remains unidentified. Graham has suggested that a subgroup of children with CHARGE Association have a recognizable syndrome [5] and may have a common pathologic and molecular basis. We have focused our study in this subset of patients and hypothesized that within this group, there is a common chromosomal region where recombination events lead to frequent microdeletion. In a previous study we used short tandem repeat (STR) markers spaced at an average of 5 cM to examine ten CHARGE case-parent trios for a large common deletion [6]. STR markers, because they are multi-allelic, generally are highly informative in each trio and there is little ambiguity regarding Mendelian transmission of parental alleles. The study did not identify any common deletion, but it was limited because of the relatively broad marker spacing. We have now extended the analysis by genotyping 3258 diallelic SNP markers with an average spacing of approximately 800 kb, in the same nuclear families. If microdeletion is the underlying basis of this disorder, then genotyping using this dense set of SNPs would be expected to uncover loci with loss of expected heterozygosity in the probands. We have taken into account the major and minor allele frequencies of each of the 3258 SNP markers and used a metric called the expected deletion detection, EDD [Belmont *et al.*, personal communication] to evaluate specific chromosomal intervals for the probability of detecting a deletion in the sample set. Although no deletion was detected in the CHARGE study sample, we conclude that this method could be generally useful in other studies in which small deletions occur as part of the allelic spectrum of disease.

## Methods

### Patients

The diagnosis of CHARGE syndrome was established by examination by a participating clinical genetics specialist (CB, JWB, SRL). A medical history questionnaire was completed by the parents or by direct interview. For the core genotyping, 8 Caucasian and 1 Hispanic case/parent trios were selected based on the presence of 4 major criteria or three major and three minor criteria for the syndrome [7]. There were five affected males and four affected females, with ages ranging from five to twenty years. Blood was collected and transformed cell lines were established for these families. This research protocol was reviewed and approved by the Baylor College of Medicine Institutional Review Board.

### Genotyping

Genotyping for this study was carried out on an Orchid BioSciences SNPstream UHT platform (Princeton, NJ) as previously described [8]. For this study, an initial set of ~4,200 T/C SNPs were identified and selected from the public databases for incorporation into a genome wide SNP panel. After selection, the complete set of SNPs was arranged into ~350 unique 12-plex reactions for the purpose of performing the assays on the UHT platform. The complete set of markers was then validated on a set of 5 CEPH pedigrees (40 individuals) and in 3 independent populations. A final set of 3258 markers was chosen from these combined datasets for analysis after eliminating SNPs that performed poorly through all populations, SNPs that failed both Hardy-Weinberg and Mendelian error calculations and any SNPs that were not polymorphic in the evaluated populations. An average genome wide spacing of ~800 kb between markers was achieved for this panel.

This genotyping method utilized Orchid's single base primer extension chemistry (SBE) to identify which bases were present at the site of interrogation. Multiplexed reactions (12-plex) were performed in a single tube that incorporated labeled chain terminating nucleotides onto the ends of the SBE oligonucleotides. These reactions were then hybridized onto a microarray format, that facilitates the solid-phase sorting of the labeled extension-primers to a set of universal tagged primers arrayed on the surface of the plate. The universal tags were arranged on the surface of the microarray plate in a 384-well microwell layout. This microarray format created a generic design consisting of 384 4 × 4 arrays that contained 12 oligonucleotides that corresponded to 12 unique universal capture tags. The four additional oligonucleotides, plotted in each array, were used for positive and negative controls. Genotyping calls were determined by the presence or absence of incorporated dyes that appeared at each spot on the printed arrays.

### TaqMan polymerase chain reaction

Two Assays-on-demand SNPs, rs422951 and ss1309424, flanking rs431722 were obtained from Applied Biosystems and genotyping was performed in 384 well-plates, using the TaqMan polymerase chain reaction-based method. The final volume reaction was 5 μl using 12 ng of genomic DNA, 2.5 μl of Taqman Master mix and 0.25 μl of 20X Assays-on-Demand SNP Genotyping Assay Mix. The plate was heated at 95° for 10 minutes, followed by forty cycles of denaturation at 92° for 15 seconds and annealing/extension at 60° for 1 minute. PCR plates were read on ABI PRISM 7900HT instrument with SDS v2.0 software. Individual genotypes that were ambiguous were excluded.

### Fish

Bacterial Artificial chromosomes (BACs) were selected from the public database [9] and obtained from Children's Hospital Oakland Research Institute. DNA extraction was performed according to the standard protocol. Fluorescence *in situ *hybridization was performed as described elsewhere [6]. Detection of the digoxigenin labeled probe was performed with anti-digoxigenin conjugated to rhodamine, giving a red signal. Biotin labeled control probe was detected with FITC (fluorescein isothiocyanate), giving a green signal. The chromosomes were counterstained with DAPI and analyzed with a Zeiss Axioskop fluorescence microscope equipped with appropriate filter combinations. Approximately 10 metaphase preparations were scored for each hybridization.

### Data analysis

The genotype error rate was estimated using the method of Gordon *et al. *[10] and as implemented in CUE [11]. Expected deletion detection (EDD) is a new method designed for this study, which uses the allele frequency, the marker spacing and the number of pedigrees sampled to estimate the probability that a common deletion would be missed because of ambiguous genotype outcomes. Qualitatively, the information available in a single SNP marker for the purposes of detecting a deletion by lack of expected heterozygosity in a case-parent trio is limited by the many genotype configurations that could appear consistent with Mendelian inheritance, but actually harbor a deletion. Inclusion of 2 or more SNP markers in a deletion interval decreases the likelihood that a common deletion goes undetected.

## Results

**Figure 1:**
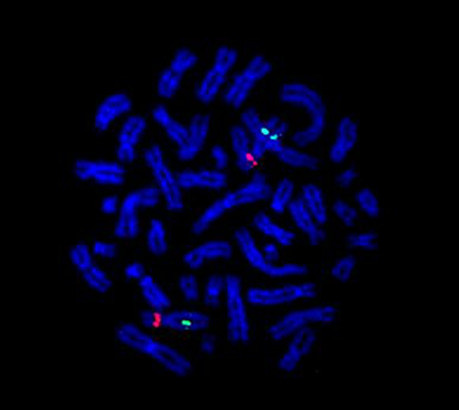
**FISH analysis of *NOTCH4 *gene**. Two copies of the locus on 6p21.32 using XXbac-300A18 clone.

SNP genotyping data were subjected to analysis for Hardy-Weinberg equilibrium. As an additional test of marker integrity, a transmission disequilibrium test was also performed for each of the SNP markers to examine for distortions in allele transmission in the trios. Unequal transmission of alleles from the heterozygote parents to the affected offspring was not determined by this analysis. Non-paternity was excluded in core pedigrees. Using the method of Gordon [12], we used the genotyping data to estimate the genotyping error rate at 0.02%. Analysis of the data showed transmission inconsistent with Mendelian inheritance for 22 markers on different chromosomes (20 with 1 inconsistency, 1 with 2 inconsistencies, and 1 with 4 inconsistencies). Given the underlying genotyping error rate, we could predict that markers with >2 inconsistencies would be highly unlikely to occur. One marker, rs431722 with overall call rate of 95%, showed Mendelian inconsistency in 4 trios. This SNP was found to lie within the intron 2 of the *NOTCH4 *gene on chromosome 6p21.32. Human DNA sequence from clone XXbac-300A18 (GenBank accession number AL662884) on chromosome 6p21 was used for FISH. This BAC clone encompasses the *NOTCH4 *gene, and was confirmed by PCR amplification of the clone sequence using *NOTCH4 *specific primers (data not shown). The analysis of the metaphase chromosomes after staining with DAPI showed two bright hybridization signals, indicating the presence of both alleles (Figure 1). Two additional SNPs, rs422951 and ss1309424, flanking rs431722 within 780 bp, were genotyped using TaqMan chemistry. The results showed inheritance of biparental alleles in all four pedigrees. The *CHD7 *locus on 8q12 was investigated additionally with FISH using RP11-33I11 (GenBank accession number AC113143) and RP11-414L17 (GenBank accession number AC023102). Using these BAC clones, microdeletion of this region was excluded in all the affected patients in this study sample.

**Figure 2:**
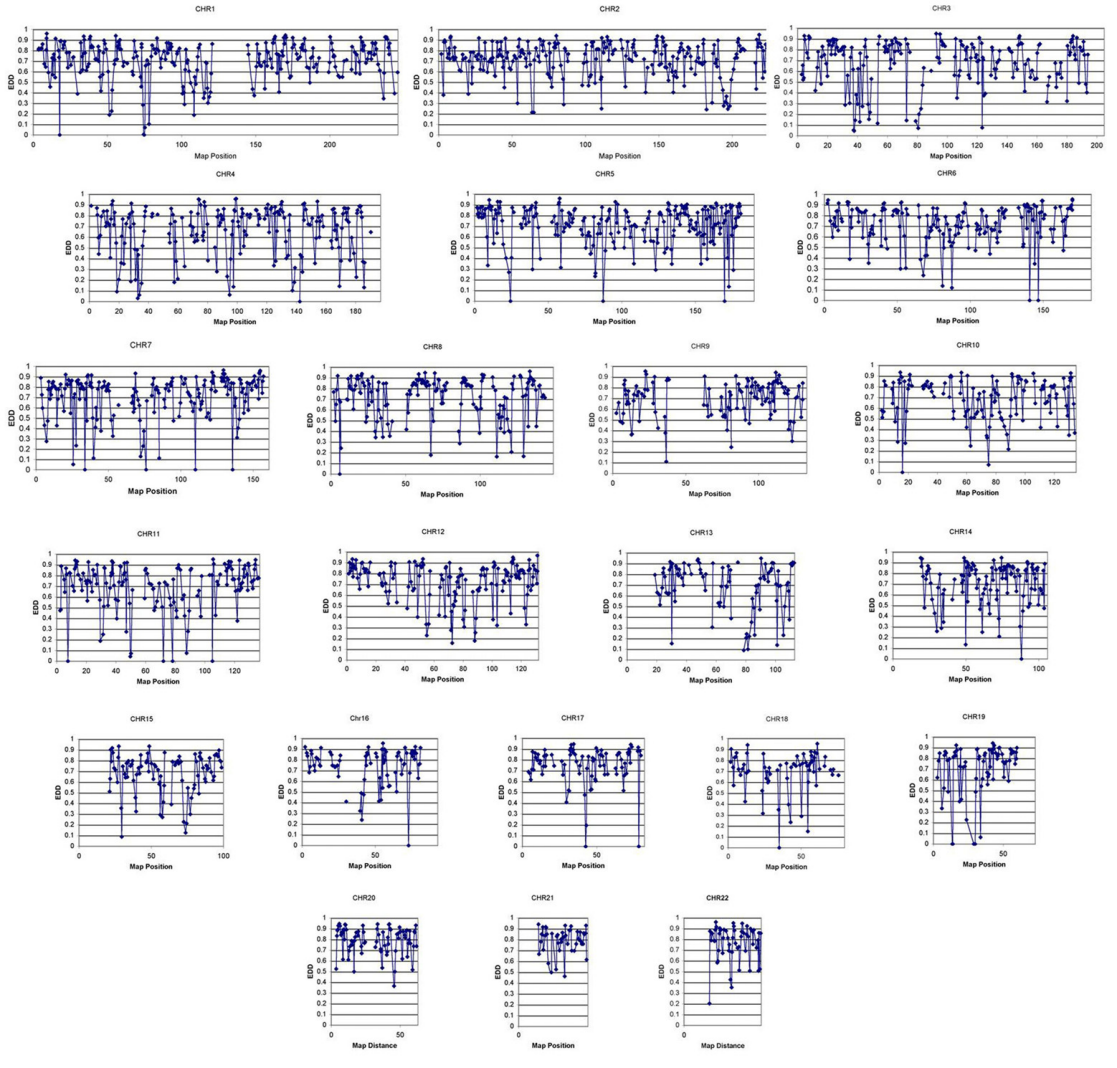
**Deletion coverage**. Chromosome-specific maps indicating the Expected Deletion Detection over each interval.

Because none of the pedigrees were consanguineous, we used parental data to estimate the allele frequencies for each of the 3258 SNP markers [13]. The EDD was then calculated for each chromosome. The percent coverage ranged from 51% on chromosome 20 to 20% on chromosome 15, with a mean of 36% for an autosomal deletion of 2 Mb (Figure 2).

## Discussion

Despite various efforts to understand the molecular basis of CHARGE syndrome, with candidate genes sequencing [14,15], comparative genomic hybridization [16], and genome-wide scan for microdeletion(s) using microsatellite markers [6], the underlying molecular mechanism in many patients remained unknown until recently [4]. Based on the complex phenotype and clinical overlap with Velocardiofacial syndrome, it is a plausible hypothesis that in a subset of CHARGE patients with a homogenous phenotype, the underlying genetic mechanism is a cryptic submicroscopic deletion involving highly pleiotropic gene(s). To address this hypothesis, we had previously used microsatellite markers to ascertain loci with loss of expected heterozygosity in case-parent trios. SNPs are far more abundant than microsatellite markers but have not yet been used extensively in linkage and loss of heterozygosity (LOH) studies. The present study represents the application of SNPs to scan for potential submicroscopic deletions across the autosomes. Amos *et al. *have previously shown that SNP genotyping of child-parent trios provides valuable information about the presence of *de novo *microdeletion when sufficient families are studied [17]. However, this method is most appropriate when linkage disequilibrium is accounted for because of high SNP marker density. They have provided a general analytical framework and point out the effects of non-paternity, sample mix-up, and genotyping error in the interpretation of Mendelian inconsistency in case-parent trio data with biallelic markers. As expected, increases in rates of such phenomenon in the data decrease power to detect a microdeletion. In addition, they point out that heterogeneity in the position of a putative deletion also has a large impact on power. Their analysis is particularly apt given the probable future availability of extremely high density SNP marker maps and the technical capability to genotype hundreds of thousands of markers per research subject. However, they do not explore the effect of intermarker distance in the ability to detect deletions of various sizes.

Assuming the genotyping error rate of 0.5%, the probability of observing >2 inconsistencies per marker is very low. The SNP marker rs431722 showed Mendelian inconsistency in four of nine pedigrees, with apparent loss of a parental allele in each case i.e. a frequency much higher than expected for the genotyping error rate. Interestingly, this SNP is located within the *NOTCH4 *gene on chromosome 6p21.32. The Notch gene family encodes highly conserved transmembrane receptors that are involved in intercellular signaling. The Notch signaling pathway plays an essential role in regulating embryonic vascular morphogenesis and remodeling [18]. Moreover, disruption of Notch signaling via mutation in the Notch ligand *JAG1 *is known to result in Alagille syndrome [19], nonsyndromic Tetralogy of Fallot (TOF) [20] and possibly nonsyndromic biliary extrahepatic atresia [21]. Since TOF is a heart defect commonly seen in CHARGE Syndrome, *NOTCH4 *was further studied with FISH as well as flanking SNP markers for microdeletion. The results, however conclusively excluded a discernible microdeletion at this locus.

This screen is expected to detect deletions of about 1–2 Mb depending on the overlap of the SNP markers with the deletion interval. Variable coverage for each chromosome was determined for approximately 2 Mb microdeletion in this study. Almost 50% of chromosomes 7, 19 and 20 were excluded for any microdeletion greater than 2 Mb. The least coverage was observed for chromosome 15 and 18, with exclusion of 20% of the chromosome for the presence of a similar genetic aberration. Overall, we can estimate that approximately 36% of the genome had >80% chance for detecting a common 2 Mb deletion in at least 2 patients with CHARGE Syndrome.

There are several limitations to this approach in studying the genetics of CHARGE syndrome. Although the marker density is high, the reduced amount of information per marker means that only some of the trios give the possibility of a conclusive result. Denser marker sets would be predicted to fill most of the gaps, but the regions around the centromeres are likely to be difficult with any currently available technique.

The strategy out lined in this paper would work equally well for conventional Mendelian traits in which the mutant alleles included at least some deletions. Using a much denser SNP map of 1 marker every 5–10 kb, as is anticipated for whole genome association analyses, it would be possible to detect most deletions given a sufficient representation of deletions within the spectrum of gene mutations.

## Conclusions

In this report we show that a SNP genotyping screen has excluded moderate length submicroscopic deletions in a subset of patients with CHARGE syndrome. Further analysis by microarray comparative genome hybridization methods or denser SNPs will allow a comprehensive assessment of the role, if any, of microdeletions in CHARGE syndrome.

## Competing interests

The author(s) declare that they have no competing interests.

## Authors' contributions

SRL carried out the data analysis, FISH and TaqMan assays and drafted the manuscript. AMS assisted in the data analysis. MP carried out the SNP genotyping, JWB conceived of the study, and participated in its design and coordination. He performed the statistical analysis and drafted the manuscript. SDF, CAB, and NLG participated in patient enrollment and LMM assisted in DNA extraction. WJC and JAT assisted in manuscript preparation. All authors read and approved the final manuscript.

## Pre-publication history

The pre-publication history for this paper can be accessed here:
